# Supplementary material for: Pathways controlling neurotoxicity and proteostasis in mitochondrial complex I deficiency
Source: Hum Mol Genet. 2024 Feb 7;33(10):860–71. doi: 10.1093/hmg/ddae018 (PMC11070137; doi:10.1093/hmg/ddae018)
Supplement: Supplemental_Figures_ddae018 [file supplemental_figures_ddae018.docx]

**Supplementary Figure 1. Control brains are well-preserved at 30 days of age.** Hematoxylin and eosin-stained sections of brains from control flies at 1 and 30 days of age demonstrate overall maintenance of brain and retinal structure with age. Genotype (control) is *elav-GAL4/+.* Flies are the indicated ages.

**Supplementary Figure 2. Retinal degeneration with knockdown of NDUFV1 with age.** Hematoxylin and eosin-stained sections of retinas from flies with retinal knockdown of NDUFV1 by three independent RNAi lines showing retinal degeneration with age. Scale bar is 50 µm. Control is *GMR-GAL4/+.* Flies are the indicated ages.

**Supplementary Figure 3. Autophagic and lysosomal changes in the brains of flies with pan-neuronal NDUFV1 knockdown.** (A) Representative images from flies expressing the tandem reporter GFP-mCherry-Atg8a with and without pan-neuronal knockdown of NDUFV1. (B) Quantification shows an increase in the GFP to mCherry fluorescence ratio in the brains of pan-neuronal NDUFV1 RNAi-expressing flies, indicating impaired autophagic flux. n = 30 puncta from 3 animals per genotype. Control is *elav-GAL4/+; UAS-GFP-mCherry-Atg8a/+* in A and B. (C) Images show lysosomes stained with LysoTracker. (D) Quantification shows an increase in stained lysosomes in the brains with pan-neuronal NDUFV1 knockdown. n = 6. (E) Real-time PCR shows an increase in *Atg4a* transcript levels with pan-neuronal NDUFV1 knockdown. n = 3 repeats with 10 flies per repeat. Control is *elav-GAL4/+* in C, D and E. Data are represented as mean ± SEM. *p<0.05, **p<0.01, ***p < 0.001, t-test. Scale bars are 2 μm in A and 5 μm in C. Flies are 10 days old.

**Supplementary Figure 4.** **Increased oxidative stress with retinal NDUFV1 knockdown.** (A) Immunostaining for GFP in retinas from flies expressing *GstD1-GFP*, an oxidative stress reporter, in the retina with and without retinal NDUFV1 knockdown. Scale bar is 20 μm. (B) Quantification shows the increase in oxidative stress reporter levels due to NDUFV1 knockdown. n = 6 per genotype. Control is *GMR-GAL4/+; GstD1-GFP/+*. Flies are 10 days old. (C – F) Western blot (C, E) and quantification (D, F) shows increased Atg8a-II (C, D) and p62 (E, F) in pan-neuronal NDUFV1 knockdown fly heads. n = 3 per genotype. Control is *elav-GAL4/+.* Flies are 30 days old. All data are represented as mean ± SEM. *p<0.05, **p<0.01, ***p < 0.001, t-test.
